# Comparative effects of pollen limitation, floral traits and pollinators on reproductive success of *Hedysarum scoparium* Fisch. et Mey. in different habitats

**DOI:** 10.1186/s12870-021-03211-2

**Published:** 2021-09-18

**Authors:** Min Chen, Xue-Yong Zhao, Xiao-An Zuo, Shao-Kun Wang, Hao Qu, Yue Ping, Xu-Jun Ma, Liang-Xu Liu

**Affiliations:** 1 Northwest Institute of Eco-Environment and Resources, Chinese Academy of Sciences, Lanzhou, 730000 China; 2 Urat Desert-grassland Research Station, Northwest Institute of Eco-Environment and Resources, Chinese Academy of Sciences, Lanzhou, China; 3 Naiman Desertification Research Station, Northwest Institute of Eco-Environment and Resources, Chinese Academy of Sciences, Lanzhou, China; 4 Key Laboratory of Stress Physiology and Ecology in Cold and Arid Regions, Gansu Province, Lanzhou, 730000 China

**Keywords:** Pollen limitation, Floral traits, Pollinators, Seed set, Fragmented habitat

## Abstract

**Background:**

Reproduction in most flowering plants may be limited because of the decreased visitation or activity of pollinators in fragmented habitats. *Hedysarum scoparium* Fisch. et Mey. is an arid region shrub with ecological importance. We explored the pollen limitation and seed set of *Hedysarum scoparium* in fragmented and restored environments, and examined whether pollen limitation is a significant limiting factor for seed set. We also compared floral traits and pollinator visitation between both habitats, and we determined the difference of floral traits and pollinators influenced reproductive success in *Hedysarum scoparium*.

**Results:**

Our results indicated that supplementation with pollen significantly increased seed set per flower, which is pollen-limited in this species. Furthermore, there was greater seed set of the hand cross-pollination group in the restored habitat compared to the fragmented environment. More visits by *Apis mellifera* were recorded in the restored habitats, which may explain the difference in seed production between the fragmented and restored habitats.

**Conclusions:**

In this study, a positive association between pollinator visitation frequency and open flower number was observed. The findings of this study are important for experimentally quantifying the effects of floral traits and pollinators on plant reproductive success in different habitats.

**Supplementary Information:**

The online version contains supplementary material available at 10.1186/s12870-021-03211-2.

## Background

Pollen limitation, a decrease in potential plant reproduction due to inadequate pollen receipt, is ubiquitous across Angiosperms [1, 2]. Most flowering plants are moderately to highly generalist in their pollination system, typically being visited by a variety of pollinators that may or may not differ in their reproduction effectiveness [3]. The floral traits of flowering plants may have evolved to attract and utilize a relatively large number of pollinators [4]. Moreover, floral trait evolution under pollinator selection is associated with incredibly specialized adaptive pathways as a result of more effective pollinator visitation in pollination syndromes [5]. These floral traits might both facilitate pollination by the primary pollinator and impede other possible pollinators, which could enhance the transfer of pollen [3].

Plants are immobile and therefore they are dependent on abiotic or biotic vectors for the transportation of pollen for sexual reproduction [1]. In the sexual reproduction of flowering plants, pollination is the initial stage and impacts numerous ecological and evolutionary processes [6–8]. Hadley and Betts suggested that the destruction of pollination mutualisms as a result of habitat loss and fragmentation can be attributed to damaged pollinator pools and restricted pollinator movement, and as a consequence, a decline in pollen transfer among the fragmented habitats [9]. As one of the greatest sources of biodiversity loss, habitat fragmentation significantly affects plant reproductive success [10, 11]. In animal-pollinated plants, pollen limitation seems to be associated with decreased pollinator abundance, diversity, and activity [2, 12]. Many reviews have shown that fragmented habitats can alter the foraging patterns of pollinators, which influences their success as pollinators, thus altering sexual reproduction in the plants [13–15]. Rodríguez-Oseguera et al. demonstrated that pollinator frequency and success may differ among populations in accordance with their state of disturbance [14]. Arias-Cóyotl et al. reported that variation in pollinator visits between wild and cultivated populations of *Stenocereus stellatus*, and the consequent effects on reproductive success were likely a result of differences in foraging and in sensitivity of pollinators to cultivated populations [16].

The fragmentation of habitats causes isolation, edge effects, and disturbed species interactions, for instance, plant-animal mutualisms [17]. Steffan-Dewenter indicated that the abundance and activity of pollinators decreases in fragmented habitats as a result of either reduced floral rewards or the inability of the habitat to satisfy the nesting requirements of the pollinators [18]. Corbett reported that pollinator behavior, the pollen quality and quantity supplied to each stigma, and pollen delivery are biotic factors with significant influences on effective reproduction [19]. In addition, many self-incompatible plants are particularly vulnerable to the reduction of pollinators in fragments due to their major reliance on pollinators for reproduction [20, 21].

*Hedysarum scoparium* plays a critical role in the establishment of arid vegetation. The root system of *H. scoparium* is well-developed and drought resistant. It is a species with wind resistance and sand-fixation characteristics, and thus plays an important role in the maintenance of arid ecosystem stability. Their leaves has great potential for livestock feed. This species occurs primarily in the arid regions of western Inner Mongolia, Ningxia, and Gansu in China [22]. In addition, *H. scoparium* is self-compatibility and pollinator pollination played a critical role in breeding system [23]. This species was severely disturbed by human activities, and it is now fragmented and isolated. Furthermore, little attention has been given to how restored habitats affect reproductive success in this species. In the present study, we aimed to evaluate how pollen limitation and pollinator activity impact seed set, assessing whether an altered habitat influences pollen limitation, floral traits, and pollinator visitation in *H. scoparium*. Our primary aims were to: 1) evaluate the relative impact of pollen supplementation on seed set and establish the potential variation in pollen limitation between fragmented and restored habitats; 2) examine how the restored habitat affects pollinator activity and visitation frequency; and 3) study the effect of floral traits and pollinators on seed production in different habitats.

## Results

### Pollen limitation

**Fig. 1:**
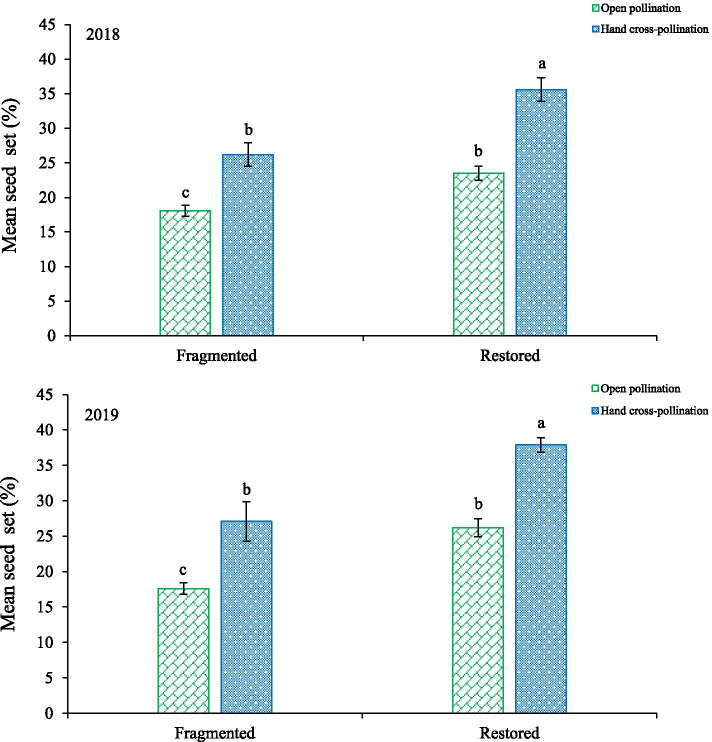
Mean seed set in the pollination treatments (mean ± SD). Different letters show a significant difference at the 0.05 level

In the fragmented habitat, the seed set averaged 18.1 ± 0.8% (2018) and 17.6 ± 0.8% (2019) in the open pollination group, and 26.2 ± 1.0% (2018) and 27.1 ± 1.3% (2019) in the hand cross-pollination group. In the restored habitat, the seed set of open pollination group was 23.5 ± 1.7% (2018) and 26.2 ± 2.8% (2019), while the seed set of hand cross-pollination group was 35.6 ± 1.7% (2018) and 37.9 ± 1.0% (2019) (Fig. 1). Our results indicated that pollen supplementation and location in a restored habitat significantly increased the seed set (GLM, pollen addition treatment effect: likelihood ratio^2^ = 56.34, *df* = 1, *P* < 0.001; Table 1). This suggested that pollen limitation is a significant limiting factor for seed set. In addition, we obtained a pollen limitation index of 0.33 in the fragmented habitat and 0.32 in the restored habitat. Given these pollination limitation index values, there are insignificant differences between the studied habitats. According to our results, the reason is that the S_OP_ and S_CP_ are both different between fragmented habitat and restored habitat.

**Table 1:** Impact of habitat types (restored and fragmented), treatments (OP and CP) and years (2018 to 2019) on the seed set of *H. scoparium*. OP, open pollination treatment; CP, hand cross-pollination treatment

	Seed set		
	likelihood ratio^2^	*df*	*P*
Habitat types	56.340	1	P < 0.001
Treatments	65.128	1	P < 0.001
Years	3.813	1	0.51

### Pollinator visitation and activity

**Fig. 2:**
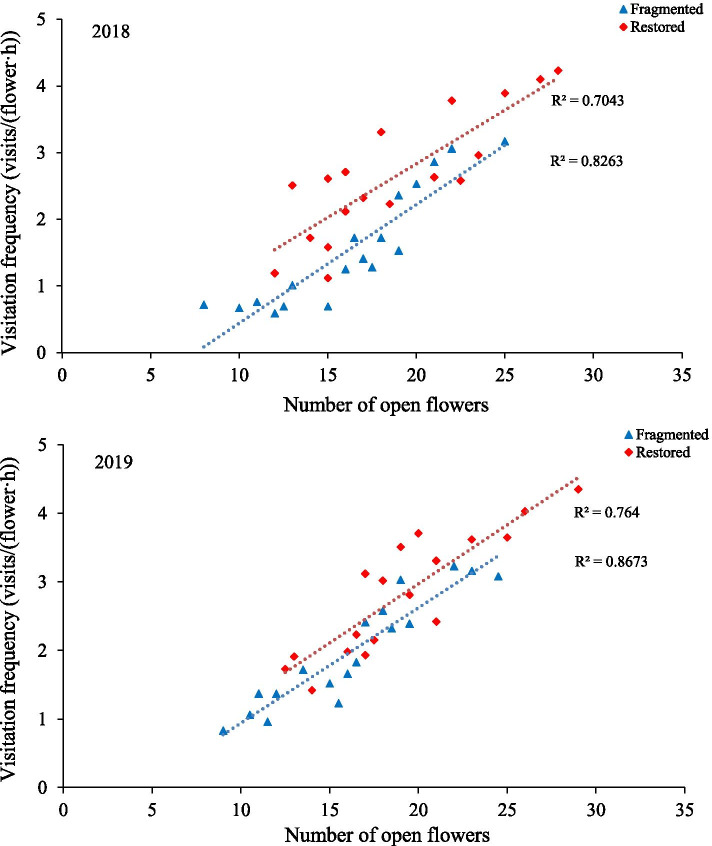
The relationship between pollinator visitation frequency and open flower numbers in the studied habitats

The *H. scoparium* flowers attracted a high diversity of pollinators, including bees (Hymenoptera) and butterflies (Lepidoptera). Individuals of *Apis mellifera* ligustica Spinola were observed significantly more frequently than other pollinators (*P* < 0.05). In addition, *Megachile spissula* Cockerell, *Anthophora deserticola* Morawitz, *Amegilla (Zebramegilla) salvia* (Morawitz), and *Pieris rapae* Linne were commonly observed. During the visiting process, flowers were intensively visited mainly by *A. mellifera* (4.3 visits per hour), and other pollinators showed visitation frequencies lower than 1.8 visits per hour. In addition, the highest V_f_ of *A. mellifera* was 3.2 ± 1.0 in the fragmented habitat and 4.3 ± 1.5 in the restored habitat, and the differences in the V_f_ values for the two habitat types was significant (*P* < 0.05). These findings suggest that the pollinator visiting frequency was significantly correlated with open flower number (the percentage of seeds: r = 0.91, 2018; r = 0.93, 2019; *P* < 0.01) in the fragmented and restored habitats (the percentage of seeds: r = 0.84, 2018; r = 0.87, 2019; *P* < 0.01; Fig. 2).

**Fig. 3:**
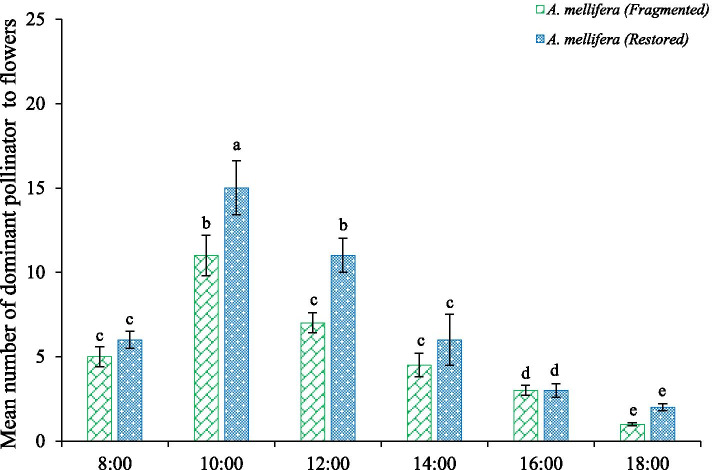
Dominant pollinator activity of the *H. scoparium* flowers across the different habitats

The *H. scoparium* flowers have a tripping mechanism whereby pollinator acts as tripping agents. *Apis mellifera* landed on the front of the flower, using its head to drive the flap forward while tripping the flap with its forefoot. It inserted its beak into the base of the petals to obtain pollen and nectar. The majority of the pollinators landed on the corolla lobes and brushed the stigma and stamens. The *A. mellifera* visits were greatest from 10:00 to 16:00, which coincided with the complete release of the pollen (Fig. 3). This activity may account for the greater ability of *A. mellifera* to transport and deposit pollen and nectar in comparison to the other pollinators.

### Floral and vegetative traits

The plant heights, numbers of flowers, corolla sizes, and spur lengths that were obtained for fragmented and restored habitats are shown in Table 2. We found that corolla size and spur length were not significantly different between the treatments, whereas the plant height and number of open flowers were significant higher in the restored habitat than the fragmented habitat (Table 2). Our observations in *H. scoparium* indicated that the majority of flowers started to open at approximately 08:00, being completely open by about 09:00. The average open flower number of each plant in the restored habitat (14.6 ± 2.6) was significantly greater than in the fragmented habitat (10.2 ± 2.1; *df* = 1, *P* < 0.05; Table 2).

**Table 2:** Floral traits (mean ± SD) of *H. scoparium* in restored and fragmented habitats

Traits	Fragmented	Restored	*P*
Plant height (cm)	171.3 ± 23.6	197.6 ± 20.1	*P* < 0.05
Number of open flowers	10.2 ± 2.1	14.6 ± 2.6	*P* < 0.05
Spur length (mm)	9.2 ± 1.5	9.7 ± 21.8	*P* > 0.05
Corolla size (mm^2^)	162.3 ± 32.2	181.3 ± 26.8	*P* > 0.05

**Fig. 4:**
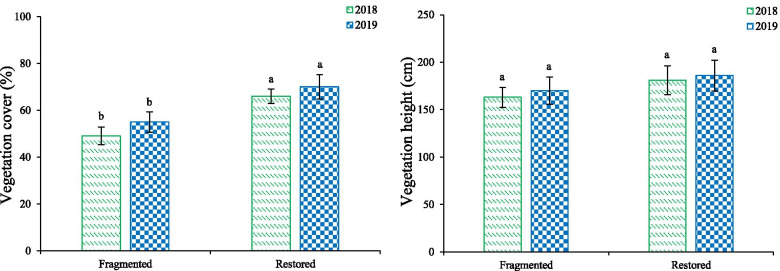
Effects of different habitats on vegetation. Vegetation cover and vegetation height of *H. scoparium*

The vegetation cover and vegetation height of *H. scoparium* are indicated in Fig. 4. According to the results, the vegetation cover was significantly higher in the restored habitat in comparison to the fragmented habitat (*df* = 1, *P* < 0.05). However, there was no difference in vegetation height between the two habitats (*df* = 1, *P* > 0.05).

### Effect of pollinator visitation on seed production

**Fig. 5:**
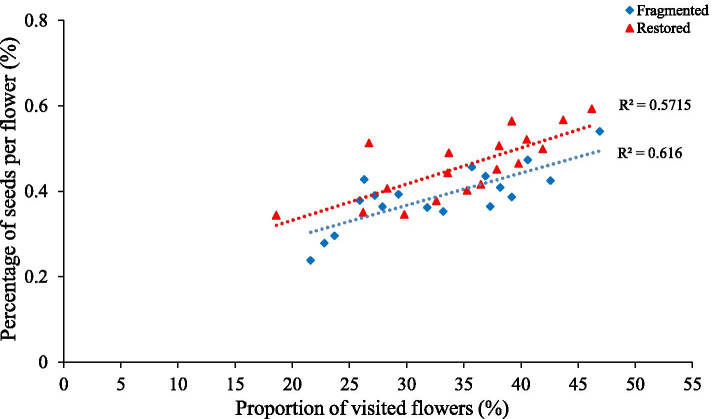
Relationship between the percentage of seeds per flower and the proportion of visited flowers for *H. scoparium*. Different letters indicate a significant difference at the 0.05 level

In the fragmented habitat, 30.23% of the flowers were visited (V) and 12.31% of the flowers produced seeds (S), indicating that the percentage of seeds among visited flowers (S/V × 100%) was 40.72%. In the restored habitat, 35.56% of the flowers were visited and 16.29% of the flowers produced seeds, resulting in a seed percentage for the visited flowers of 45.81%. These findings demonstrated that increased pollinator visitation rates increased seed production percentages in both studied habitats (Fig. 5).

## Discussion

### Pollen limitation and reproductive success

Pollen limitation takes place when incompatible pollen is deposited by pollinators or when pollinators are ineffective, and it is a widely observed phenomenon [2, 24, 25]. According to previous studies, fragmented habitats alter functional interactions, such as plant–pollinator mutualisms [26, 27]. Most flowering plant species are pollinated by many effective pollinator species, and pollinator activity over space and time adapts to the pollination process [5, 28]. There is a strong association between pollen limitation and habitat fragmentation, with the spatial and temporal effects of fragmentation further compounding reproductive success [29, 30]. In the present study, we determined that pollen limitation is a significant limiting factor for the seed set of *H. scoparium*.

Most animal-pollinated flowers are subject to pollen limitation as a result of unreliable pollinators [1, 31]. In these plants, pollinator abundance and identity are the primary causes of insufficient pollen deposition [32]. Ashman et al. showed that pollinators are generally less attracted to plant species with low-density flowers, which typically receive less pollen than species with high-density flowers [1]. In *H. scoparium*, the dominant pollinators opted to frequent the restored habitat with greater flower resource availability than the fragmented habitats, as more pollen could be collected from the floral resources. Furthermore, the higher seed set of the hand cross-pollination group was observed in the restored habitat than in the fragmented habitat.

### Pollinators and pollinator activity in fragmented and restored habitats

The decline in pollinator function as a result of increased habitat conversion is of global concern. This is particularly true in arid areas where high rates of habitat loss and fragmentation correspond with a higher proportion of angiosperms dependent on animal-mediated pollination [9]. It has been shown that pollinators have different responses to the variation in floral density in fragmented habitats [33]. A similar study found that fragmented habitats and vegetation clearance might influence flower production in *Escontria chiotilla* [34]. A study on plant reproduction in fragmented habitats suggested that pollinator activity has an important influence on reproductive success [35]. Our results indicate that pollen release by *H. scoparium* occurred from 09:00 to 15:00, which corresponded to the most critical period for pollination, and furthermore, coincided with peak visits from *A. mellifera*. Previous reviews indicated that honeybee has an outstanding role in many of the crops’ pollination, but *Apis mellifera* sometimes was not a very successful pollinator [36, 37]. Ryan and David suggested that the convergent evolution of floral traits with the traits of their dominant pollinators is widespread in plants and constitutes one of the most visual demonstrations of natural selection [38]. In arid regions, a high frequency of pollinator visits is more efficient because the filaments of flowers dry easily. In *H. ammodendron*, the flowers have a tripping mechanism. *A. mellifera* activity starts with flowers in anthesis, and the pollinator also acts as a tripping agent. In present study, compared to other pollinators, *A. mellifera* can collect more pollen and visit more flowers. Therefore, *A. mellifera* could be treated as an effective pollinator of the studied plant.

Pollinator activity could be associated with human impacts that affect pollinator visitation and behavior [39]. In animal-pollinated plants, pollination limitation has been strongly linked to habitat fragmentation [11, 21]. A lack of pollinators or reduced pollinator efficiency in many species can reduce pollination efficiency, and pollinator species richness also tends to decline with habitat isolation in fragmented areas [17, 21, 40]. The findings in the present study might explain why visiting frequency and number of open flowers are positively correlated. Kearns and Inouye showed that fragmented habitats reduce reproductive efficiency, as pollinators respond sensitively to habitat destruction in fragmented habitats [41]. Fragmented habitats cause isolation and produce conditions that can affect pollinator behavior during the reproduction process [10]. Pollinator reduction results in a decline in the quantity of pollen delivered to stigmas and decreases the probability of cross-pollination transfer in fragmented area. An increase in the spatial distance between plant populations and reduced pollinator habitat, in turn leads to reduced pollinator visitation and increased genetic isolation of plant populations [11]. The fragmented experiments showed that fragmented habitats had a significant effect on plant resources, and it significantly reduces vegetation cover and excessive human interference has been considered a significant contributor to grassland degradation [39]. Furthermore, decreased management intensity and human impacts in arid region can improve the maintenance of diverse plant-pollinator communities [37].

### Floral traits and pollinators affect seed production

Pollinators are major selective agents on floral traits [42]. Floral traits and the anthers attract pollinators to specific flowers, and an association between flower resource density and the variation in visiting frequency and pollinator behavior might exist [3, 43]. Our findings suggested that the open flowers of the restored habitat have greater floral resources than those in the fragmented habitat. Nayak and Davidar suggested that the extent of pollinator dependence is chiefly determined by plant reproduction, while Aguilar et al. noted that pollinator visitation can affect the pollination success of plants in fragmented areas [21, 35]. Pollinator activity is the effective pollination model, and a reduction in pollinators reduces the probability of cross-pollen transfer [44]. If pollinators are inadequate and pollen limitation occurs, the loss of pollinator activity could potentially threaten the pollination success of plants [21].

Variations in visiting frequency and pollinator behavior might be correlated with flower resource density [43]. For instance, floral traits as well as the anthers lure pollinators to particular flowers, with the targets of the pollinators being nectar and pollen. The restored habitat had a greater flower density than the fragmented habitat in our study, which might account for the variation in pollinator visitation between the both studied habitats. In *H. scoparium*, *A. mellifera* visited regions with greater resource abundance and spent more time in the restored habitats. Furthermore, we discovered that there is an association between increased pollinator visitation and increased seed production.

## Conclusions

In the present study, pollen limitation was determined to be the dominant factor limiting seed production. There were more open flowers with large corollas and long spurs in the restored habitats than the fragmented habitats. In addition, open flowers number and pollinator visitation frequency were positively correlated. Elucidating the relative contributions of habitat type is necessary for developing effective management approaches for limiting pollination and pollinator decline.

## Methods

### Plant species

The shrub *H. scoparium* is typically 0.8 to 3.0 m in height, with 15–30 mm long and 3–6 mm broad leaflets. The flowers are purple, and the petal length is 15–20 mm. Flowers in anthesis usually occurs from June until September, while fruiting takes place between August and October [45].

### Study site and experimental layout

The experiment was performed from May 2017 to October 2019 at various sites at the Linze Inland River Basin Comprehensive Research Station in Gansu Province, China (100°07'E, 39°21'N). The area has an annual mean rainfall of approximately 118.4 mm.

**Fig. 6:**
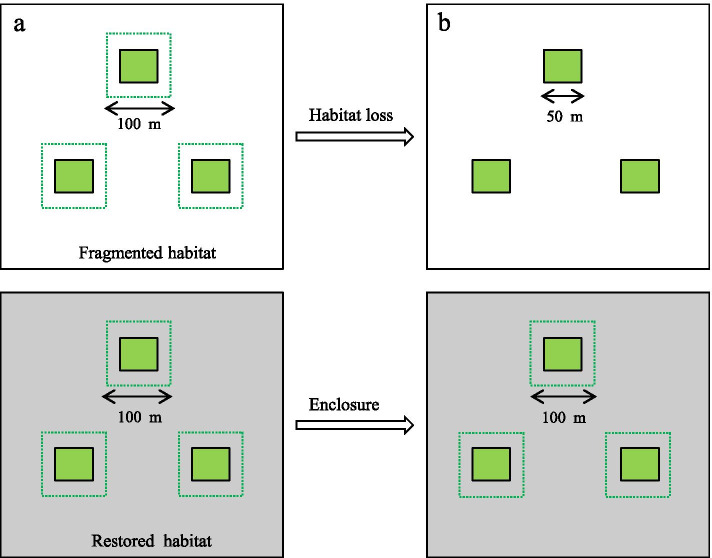
Experimental layout of the two habitats from 2017 to 2019; one represents the fragmented habitat and the other represents the restored habitat. Both the fragmented and restored plots were separated by mown vegetation (white area), which were mirrored in a symmetrical arrangement and were enclosed by undisturbed vegetation (gray area). **a** Plot size of habitats (plots were 100 × 100 m) in 2017; **b** Plot size of habitat (fragmented plots were 50 × 50 m; restored plots were 100 × 100 m) in 2019

The experimental layout comprised a total of two habitats and six plots (three fragmented plots and three restored plots). The fragmented habitat was situated in an arid area where the topsoil had been eroded. Furthermore, the three plots in the fragmented area were separated by vegetated regions that were regularly mown. The corresponding restored habitat possessed the same arrangement and plant community as the fragmented habitat (Fig. 6). The *H. scoparium* was planted in the restored habitat in 2017 after enclosure and soil conservation, and this habitat was protected from livestock grazing and other human impacts. The fragmented and restored habitats were separated by nearly 2 km to reduce mutual pollinator interference. The mean plant density was 10 individuals per 100 m^2^, and each plot was separated by 200 m. Furthermore, the different inflorescences in the same six marked plants per plots were used for these experiments such as pollen limitation, pollinator visitation frequency, and floral traits.

In addition, the seeds of *H. scoparium* were originally acquired from the Lab of Linze Inland River Basin Comprehensive Research Station. In these experimental research on plants, including collection of plant material, we comply with institutional, national, or international guidelines. The field studies also conducted in accordance with local legislation.

### Pollen limitation

To evaluate if pollen limitation impacts reproductive success, we evaluated the seed set in open pollination and hand cross-pollination treatments from 2018 to 2019. In hand cross-pollination, the fresh pollen was collected from five to ten flowers, and these flowers were from a different plant located a minimum of 20 m away. In each habitat, we randomly marked 18 flowering plants (six plants per plot) at the budding stage. To evaluate the impacts of habitat types (fragmented and restored) and treatments (open pollination and hand cross-pollination) on seed set, we randomly allocated nine plants to the open pollination treatment and the other nine plants to the hand cross-pollination treatment in each habitat [46]. Moreover, we selected 10 flowers on each marked plant to keep open to avoid the potential mixed effects of resource reallocation. We collected and recorded seed production from early September to mid-October when all of the seeds had matured. We estimated pollen limitation according to the seed set from the hand cross-pollination (CP) and open pollination (OP) treatments based on Larson and Barrett [47]:

$$\mathrm{PL}=1-\left({\mathrm{S}}_{\mathrm{OP}}/{\mathrm{S}}_{\mathrm{CP}}\right).$$

Positive values denote greater reproductive success in the CP treatment group in comparison to the OP treatment group, thus indicating pollinator limitation. Conversely, zero or negative values indicate no pollen limitation.

### Pollinator visitation and activity

To understand the association between pollinator visitation frequency and open flower number, six flowering plants from each plot were selected for observation. A flowering branch with three inflorescences was randomly chosen on each labeled plant, from which the pollinators that contacted the anthers and stigma of the flowers were recorded. Moreover, the activity of pollinators was determined, and we used HD camera to measure pollinator visit duration. We carefully analyzed the presence or absence of pollen grains adhering to the bodies of the pollinators and determined whether they contacted stamens and stigmas. We later identified the floral visitors in the laboratory. Pollen was collected by using a cube of fuchsin-stained jelly to rub the pollinator body following the Beattie’s method [48].The pollinator species with the highest visitation frequency was determined as the principal pollinator for this plant species. The following equation was used to calculate pollinator visitation frequency [49]:

$$\mathrm{Visitation}\ \mathrm{frequency}=\frac{\mathrm{NV}\ }{\ \mathrm{NF}\ast \mathrm{T}},$$

where N_V_ is the number of visits, N_F_ is the number of flowers, and T is the observation time of pollinators.

### Floral and vegetation traits

To calculate the number of open flowers in the two habitats, six flowering plants (three inflorescences per plant) were labelled in every plot. Plant height was measured to the nearest mm (distance from the ground to the topmost flower) in the late flowering period in each labelled plant. The spur length (distance from corolla to spur tip) and the corolla size (the product of width and height) were measured to the nearest 0.1 mm using digital calipers in three selected inflorescences from each marked plant [50]. Floral observations were noted during two weeks, with the phases of flowering, the number of open flowers, and anthesis time documented during this period. Additionally, the vegetation cover and vegetation height of *H. scoparium* were also measured in the various habitats. These measurements were performed each year (from 2018 to 2019).

### Effect of pollinator visitation on seed production

To evaluate the impact of pollinator visitation on seed production, three plants per plot were marked during flowering in the different habitats. Ten flowers were randomly selected from each plant and marked with tags. The flowering progress of the marked flowers was determined (n = 180 flowers) from the HD camera. Additionally, pollinator visitation and seed production were investigated from May to September. We covered all of the flower after pollinator visiting with bags to restrict other insect visits and wind pollination, and we also calculated the pollinator visiting using all flower visitors. Moreover, we recorded the number of seeds in the bagged fruit, and the number of seeds in the mature fruits was used as an indicator of pollination success. We calculated the seed percentage among visited flowers using the following equation [6]:

$$\mathrm{Percentage}\ \mathrm{of}\ \mathrm{seeds}\ \mathrm{among}\ \mathrm{visited}\ \mathrm{flowers}=\frac{\mathrm{S}}{\ \mathrm{V}} \times 100\%,$$

where V is the proportion of visited flowers and S is the proportion of seeding flowers.

### Data analyses

We used a generalized linear model (GLM) to determine the effects of pollination treatments, habitat types (fragmented and restored), and years (from 2018 to 2019) on the seed set. A gamma distribution and a logit link function were used in the model. Tag number was then used as a random factor between the two habitats. Habitat types and years were used as fixed factors, while open flowers seed set was the dependent variable. A likelihood ratio test was used in the model, and the variations in habitat types were determined with Tukey’s multiple comparisons. In addition, we used GLM to determine the effects of habitat types and years on vegetation cover and vegetation height. GLM also was used to determine pollination treatments, habitat types and years affect mean seed set. One-way ANOVA was used to compare the mean dominant pollinator number, and Multi-group comparisons of the means were carried out by one-way ANOVA test with post-hoc contrasts by S–N–K test.

Regression was also used to assess the relationship between the pollinator visitation frequency and open flower number. Open flowers was used as the independent variable in the model, while pollinator visitation frequency was the dependent variable. We also used Regression to assess the relationship between percentage of seeds per flower and proportion of visited flowers. The analyses were performed using the statistical software package SPSS 22.0.

## Supplementary Information

**Additional file 1.**

## Data Availability

Please contact the corresponding author.

## References

[1] Ashman TL, Knight TM, Steets JA, Amarasekare P, Burd M, Campbell DR (2004). Pollen limitation of plant reproduction: ecological and evolutionary causes and consequences. Ecology..

[2] Gómez JM, Abdelaziz M, Lorite J, Munõz-Pajares AJ, Perfectti F (2010). Changes in pollinator fauna cause spatial variation in pollen limitation. J Ecol.

[3] Chen M, Zhao XY, Zuo XA (2018). Pollinator activity and pollination success of *Medicago sativa* L. in a natural and a managed population. Ecol Evol.

[4] Fenster CB, Armbruster WS, Wilson P, Dudash MR, Thomson JD (2004). Pollination syndromes and floral specialization. Annu Rev Ecol Syst.

[5] Johnson SD, Steiner KE (2000). Generalization versus specialization in plant pollination systems. Trends Ecol Evol.

[6] Bond WJ (1994). Do mutualisms matter: assessing the impact of pollinator and disperser disruption on plant extinction. Philos T R Soc B.

[7] Ashman TL, Morgan MT (2004). Explaining phenotypic selection on plant attractive characters: male function, gender balance or ecological context?. Proc R Soc Lond B.

[8] Kearns CA, Inouye DW, Waser NM (1998). Endangered mutualisms: the conservation of plant–pollinator interactions. Annu Rev Ecol Syst.

[9] Hadley AS, Betts MG (2016). Corridors restore animal-mediated pollination in fragmented tropical forest landscapes. Proc R Soc B.

[10] Goddard M, Dougill A, Benton T (2010). Scaling up from gardens: biodiversity conservation in urban environments. Trends Ecol Evol.

[11] Newman BJ, Ladd P, Brundrett M, Dixon KW (2013). Effects of habitat fragmentation on plant reproductive success and population viability at the landscape and habitat scale. Biol Conserv.

[12] Krebs CJ (1985). Ecology, the experimental analysis of distribution and abundance, 3rd edn.

[13] Cresswell JE (1990). How and why do nectar-foraging bumblebees initiate movements between inflorescences of wild bergamot *Monarda fistulosa* (Lamiaceae). Oecologia..

[14] Rodríguez-Oseguera AG, Casas A, Herrerías-Diego Y, Pérez-Negrón E (2013). Effect of habitat disturbance on pollination biology of the columnar cactus *Stenocereus quevedonis* at landscape-level in central Mexico. Plant Biol.

[15] Chen M, Zhao XY (2019). Effect of fragmented habitats on pollen limitation and pollinator behavior in *Caragana korshinskii* Kom. Sci Total Environ.

[16] Arias-Cóyotl E, Stoner KE, Casas A (2006). Effectiveness of bats as pollinators of *Stenocereus stellatus* (Cactaceae) in wild, managed in situ, and cultivated population in La Mixteca Baja, Central Mexico. Am J Bot.

[17] Rodríguez-Cabal MA, Aizen MA, Novaro AJ (2007). Habitat fragmentation disrupts a plant-disperser mutualism in the temperate forest of South America. Biol Conserv.

[18] Steffan-Dewenter I, Klein AM, Gaebele V, Alfert T, Tscharntke T, Waser NM, Ollerton J (2006). Bee diversity and plant-pollinator interactions in fragmented landscapes. Plant-pollinator Interactions: from Specialization to Generalization.

[19] Corbett SA (2003). Nectar sugar content: estimating standing crop and secretion rate in the field. Apidologie..

[20] Bawa KS (1990). Plant-pollinator interactions in tropical rain forests. Annu Rev Ecol Syst.

[21] Nayak KG, Davidar P (2010). Pollinator limitation and the effect of breeding systems on plant reproduction in forest fragments. Acta Oecol.

[22] Feng XK (1982). The study of Biological, Ecological Characteristics and Afforestation Technology in *Hedysarum scoparium*. J Northwest A&F U.

[23] Pan CC, Liu LD, Hou YL, Zhang L, Wang YJ, Wang YH, Wang LJ, Zhao X (2010). Flowering characteristics and Breeding System of *Hedysarum scoparium* in the Meddle Reaches of Heihe River. J Desert Res.

[24] Aizen MA, Ashworth L, Galetto L (2002). Reproductive success in fragmented habitats: Do compatibility systems and pollination specialization matter?. J Veg Sci.

[25] Pflugshaupt K, Kollmann J, Fischer M, Roy B (2002). Pollen quantity and quality affect fruit abortion in small populations of a rare fleshy-fruited shrub. Basic Appl Ecol.

[26] Ashworth L, Aguilar R, Galetto L, Aizen M (2004). Why do pollination generalist and specialist plant species show similar reproductive susceptibility to habitat fragmentation?. J Ecol.

[27] Dixon K (2009). Pollination and restoration. Science..

[28] Herrera CM (2005). Plant generalization on pollinators: species property or local phenomenon?. Am J Bot.

[29] Pauw A (2007). Collapse of a pollination web in small conservation areas. Ecology..

[30] Pauw A, Bond WJ (2011). Mutualisms matter: pollination rate limits the distribution of oil-secreting orchids. Oikos..

[31] Motten AF (1986). Pollination ecology of the spring wildflower community of a temperate deciduous forest. Ecol Monogr.

[32] Fernández JD, Bosch J, Nieto-Ariza B, Gómez JM (2012). Pollen limitation in a narrow endemic plant: geographical variation and driving factors. Oecologia..

[33] Wagenius S, Lyon SP (2010). Reproduction of *Echinacea angustifolia* in fragmented prairie is pollen-limited but not pollinator-limited. Ecology..

[34] Oaxaca-Villa B, Casas A, Valiente-Banuet A (2006). Reproductive biology in wild and silvicultural managed populations of *Escontria chiotilla* (Cactaceae) in the Tehuacán Valley, Central México. Genet Resour Crop Evol.

[35] Aguilar R, Ashworth L, Galetto L, Aizen MA (2006). Plant reproductive susceptibility to habitat fragmentation: review and synthesis through a meta-analysis. Ecol Lett.

[36] Pattrick JG, Shepherd T, Hoppitt W, Plowman NS, Willmer P (2017). A dual function for 4-methoxybenzaldehyde in *Petasites fragrans*? Pollinator-attractant and ant-repellent. Arthropod-Plant Inte.

[37] Kovacs-Hostyanszki A, Foldesi R, Baldi A, Endredi A, Jordan F (2019). The vulnerability of plant-pollinator communities to honeybee decline: A comparative network analysis in different habitat types. Ecol Indic.

[38] Ryan DBR, David AM (2013). Resource reallocation does not influence estimates of pollen limitation or reproductive assurance in Clarkia Xantiana Subsp. Parviflora (Onagraceae). Am J Bot.

[39] Chen M, Zuo XA, Zhao XY (2020). Comparative floral characters, pollinator limitation and pollination success in different habitats of *Caragana microphylla* Lam. Front Ecol Evol.

[40] Westerkamp C (1991). Honeybees are poor pollinators-why?. Plant Syst Evol.

[41] Kearns CA, Inouye DW (1997). Pollinators, flowering plants, and conservation biology. BioScience..

[42] Anderson B, Johnson SD (2009). Geographical covariation of flower depth in a guild of fly pollinated plants. New Phytol.

[43] Ortíz FE, Stoner K, Pérez-Negrón E, Casas A (2010). Pollination biology of *Myrtillocactus schenckii* (Cactaceae) in wild and managed populations of the Tehuacán Valley, México. J Arid Environ.

[44] Revel N, Alvarez N, Gibernau M, Anahí E (2012). Investigating the relationship between pollination strategies and the size-advantage model in zoophilous plants using the reproductive biology of *Arum cylindraceum* and other European Arum species as case studies. Arthropod-Plant Inte.

[45] Xu LR (1998). The Flora of China.

[46] Rathcke BJ (2000). Hurricane causes resource and pollination limitation of fruit set in a bird-pollinated shrub. Ecology..

[47] Larson BMH, Barrett SCH (2000). A comparative analysis of pollen limitation in flowering plants. Biol J Linn Soc.

[48] Beattie AJ (1971). Technique for study of insect-borne pollen. Pan-Pac Entomol.

[49] Goverde M, Schweizer K, Baur B, Erhardt A (2002). Small-scale habitat fragmentation effects on pollinator behaviour: experimental evidence from the bumblebee *Bombus veteranus* on calcareous grasslands. Biol Conserv.

[50] Sletvold N, Grindeland JM, Ågren J (2010). Pollinator-mediated selection on floral display, spur length and flowering phenology in the deceptive orchid *Dactylorhiza lapponica*. New Phytol.

