# Supplementary material for: Comparative effects of pollen limitation, floral traits and pollinators on reproductive success of Hedysarum scoparium Fisch. et Mey. in different habitats
Source: BMC Plant Biol. 2021 Sep 18;21:426. doi: 10.1186/s12870-021-03211-2 (PMC8449482; doi:10.1186/s12870-021-03211-2)
Supplement: Supplementary file 1 — Additional file 1. [file 12870_2021_3211_MOESM1_ESM.pdf]

Time	Fragmented VF-*A. mellifera*	Restored VF-*A. mellifera*	Fragmented VF-*A. deserticola*	Restored VF-*A. deserticola*	Fragmented VF-*M. spissula*
6:00-8:00	3	3.5	0.5	0.5	0
8:00-10:00	6.5	9	1.5	2.5	0.5
10:00-12:00	4	6	3	4	1
12:00-14:00	3	4	1	1.5	0.5
14:00-16:00	2	2	0.5	0.5	0
16:00-18:00	0.5	1	0	0.5	0
Mean VF	3.2	4.3	1.1	1.6	0.3

Restored<br>VF-*M. spissula*	Fragmented<br>VF-*Pieris rapae*	Restored<br>VF-*Pieris rapae*
0.5	0	0
1	0	0.5
1	0.5	0.5
0.5	0.5	1
0.5	0	0.5
0	0	0
0.6	0.2	0.4
